# Evaluation of Diagnostic Accuracy of Directly Sampled Endometrial Cytology Using ThinPrep for Endometrial Malignancies: Comparison With Existing Endometrial Liquid‐Based Cytology

**DOI:** 10.1111/cyt.13488

**Published:** 2025-04-07

**Authors:** Rie Ikemoto, Ken Tanikawa, Morio Ijichi, Miyoko Miyakawa, Kenji Yanoh, Jun Watanabe, Yutaka Nakamura, Ken Maruyama, Tadao K. Kobayashi, Hideaki Hirai, Noriyuki Furuta, Yasuo Hirai

**Affiliations:** ^1^ Department of Laboratory Medicine SRL Advanced Lab. FMA, SRL, Inc. Fukuoka Japan; ^2^ Department of Diagnostic Pathology Yame General Hospital Fukuoka Japan; ^3^ Deputy Chief of Medical Clinic Ohbuchi Clinic Miyazaki Japan; ^4^ Department of Gynecology Yame Public Hospital Fukuoka Japan; ^5^ Department of Obstetrics and Gynecology JA Suzuka General Hospital Mie Japan; ^6^ PCL Japan Pathology and Cytology Center PCL Inc. Kawagoe Saitama Japan; ^7^ Department of Pathology JA Suzuka General Hospital Mie Japan; ^8^ Educational Institution Tenri University Member of the Board Tenri Nara Japan; ^9^ Department of Diagnostic Pathology Tokyo Medical University Hospital Tokyo Japan

**Keywords:** descriptive reporting system for endometrial cytology, diagnostic accuracy, directly sampled endometrial cytology, early detection, endometrial malignancies, ThinPrep

## Abstract

**Objective:**

The aim of this study was to evaluate the accuracy of detecting malignancies by directly sampled endometrial cytology using globally adopted ThinPrep with a novel preparation technique.

**Methods:**

Medical records and reports of pathology and cytology from June 2019 to March 2022 were reviewed. We selected 112 endometrial cytology specimens using ThinPrep with the novel preparation technique, where the clinical course or pathological samples confirmed negative or positive results. Eight cytotechnologists or cytopathologists examined the cytology specimens and provided reports based on standardised criteria from the descriptive reporting system for endometrial cytology (the Yokohama System).

**Results:**

The 112 specimens were evenly smeared and well prepared, featuring high quality, with no issues hindering microscopic examination. Examiners unfamiliar with endometrial cytology using ThinPrep showed high diagnostic accuracy, demonstrating that this modality of preparation for endometrial cytology is feasible for clinical use.

**Conclusions:**

The novel preparation method using ThinPrep successfully provided high‐quality, standardised specimens. Furthermore, employing the Yokohama System enabled high accuracy in detecting endometrial malignancies, even for examiners with minimal experience with this cytological technique. This suggests that ThinPrep‐based endometrial cytology can be globally adopted with ease, potentially contributing significantly to the early detection of endometrial cancer.

## Graphical abstract

This study evaluated the diagnostic accuracy of directly sampled endometrial cytology using ThinPrep with a novel preparation method for detecting endometrial malignancies. The method yielded high‐quality cytology samples, and examiners with minimal experience demonstrated high diagnostic accuracy using the Yokohama System, supporting its global clinical adoption.

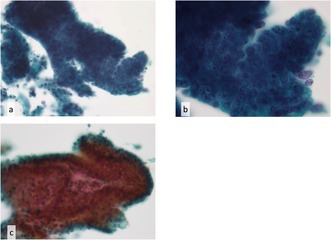

## 1 Introduction

Endometrial cytology is considered highly valuable for the early detection of endometrial cancer because it involves less invasive procedures and less discomfort than endometrial tissue biopsy [1]. However, conventional endometrial cytology specimens are challenging due to difficulties in smearing techniques in the clinical context and the high level of skill required for interpretation. As a result, conventional endometrial cytology has not been widely adopted globally [2].

Recently, several liquid‐based cytology (LBC) methods have been developed, providing standardised preparation techniques that allow for more uniform endometrial cytology specimens. Various studies have reported on the accuracy of these new methods. A prospective study by Hirai et al., using LBC (SurePath), indicated that the accuracy of endometrial cytology was non‐inferior to that of suction endometrial tissue biopsy, suggesting strongly the high accuracy and usefulness of endometrial cytology using LBC (SurePath) [3, 4, 5].

ThinPrep‐based endometrial cytology, although widely tried globally, has faced challenges in standardising preparation techniques for high‐quality samples. Recently, a groundbreaking method was developed in Japan, significantly improving the quality of endometrial cytology specimens prepared with ThinPrep [6].

This study aimed to clarify the diagnostic accuracy of directly sampled endometrial cytology using this new ThinPrep preparation method for detecting endometrial malignancies. We selected 112 cases where ThinPrep endometrial cytology samples were available and re‐evaluated these samples using the standardised descriptive reporting system (the Yokohama System) [7, 8].

## 2 Materials and Methods

### 2.1 Case Selection and Cell Sampling

Since 2019, Yame Public General Hospital has been performing directly sampled endometrial cytology using ThinPrep, with a novel preparation technique, for the early detection of uterine cancer in high‐risk patients with symptoms, such as abnormal genital bleeding. We reviewed the hospital's medical records, pathology and cytology reports from June 2019 to March 2022.

### 2.2 Sample Preparation

The samples in this study were processed using a novel specimen preparation method for ThinPrep‐based endometrial cytology. As endometrial specimens often contain a large number of blood components, which can hinder diagnosis, haemolysis was performed to remove unnecessary red blood cells as a standard work process. The following is an outline of our specimen preparation process [6, 9, 10, 11].

1. The device used to collect endometrial cells was rinsed in a ThinPrep vial.
2. The rinsed vial underwent haemolysis:
  - The entire vial content was centrifuged (1200G × 5 min).
  - The supernatant was discarded, and the sediment was mixed with 30 mL of Cytolyte Acetic Acid Solution.
  - The mixture was centrifuged again (1200G × 5 min).
  - The supernatant was discarded, and the sediment was resuspended in PreservCyt Solution. The volume was adjusted to 17–20 mL.
3. The ThinPrep processor was used to prepare the slide using a FISH‐compatible filter for UroCyto sequencing.

### 2.3 Evaluation of Cellular Sample

From these, we selected 112 cases where the clinical course or pathology samples confirmed negative or positive results, and for which endometrial cytology samples were available. These 112 cytology specimens were re‐examined by eight cytologic examiners, each of whom provided an independent cytology report based on the descriptive reporting system for endometrial cytology (the Yokohama System) [5, 7, 8, 12]. Each cytologic examiner, serving as a first screener, submitted an independent report, and the study's supervising physician at Yame Public Hospital compared the cytology results to the final pathological and/or clinical outcomes for analysis.

In total, the cytologic examiners produced 728 valid reports. The cytologic examiners consisted of seven certified cytotechnologists and one cytopathologist, all of whom had over 20 years of experience. However, they had little to no prior experience with ThinPrep‐based endometrial cytology. Each examiner used the standardised criteria from the Yokohama System to examine the specimens and submit their reports.

**FIGURE 1:**
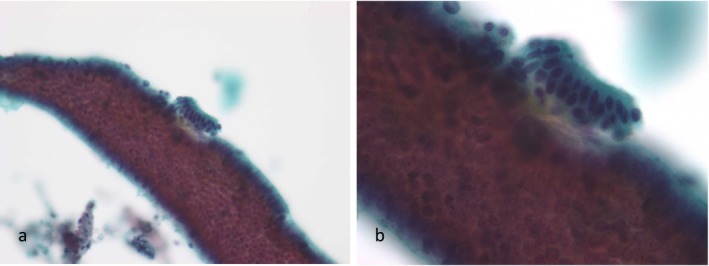
Proliferative endometrium: Straight and tubular cell clusters were observed. The width of the clusters was approximately uniform. Cell clusters composed of flat endometrial epithelial sheets were also observed (a). Endometrial stromal cells were found to adhere to the margins of these cell clusters(b). (a) ×200 magnification. (b) ×400 magnification.

**FIGURE 2:**
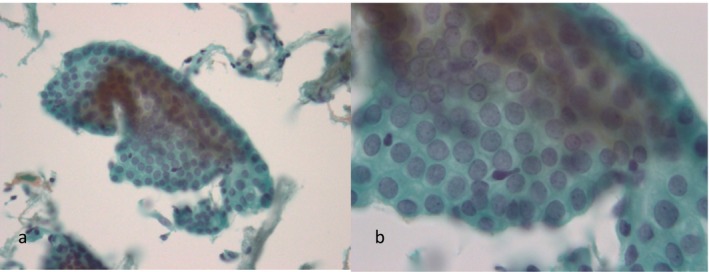
Secretory endometrium: Curved and extended tubular cell clusters were observed, as were clusters of endometrial epithelial sheets. Endometrial stromal cells were found to adhere to the margins of these cell clusters (a). In the mid‐secretory phase, a honeycomb pattern due to increased cytoplasm was observed (b). (a) ×200 magnification. (b) ×400 magnification.

**FIGURE 3:**
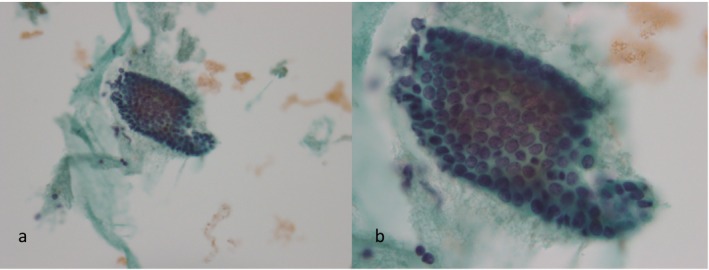
Atrophic endometrium: Small monolayered sheets were detected (a). Straight and tubular cell clusters consisting of atrophic epithelial cells were usually observed. The nuclei of these cells resembled those seen in the proliferative endometrium, but were smaller (b). (a) ×200 magnification. (b) ×400 magnification.

**FIGURE 4:**
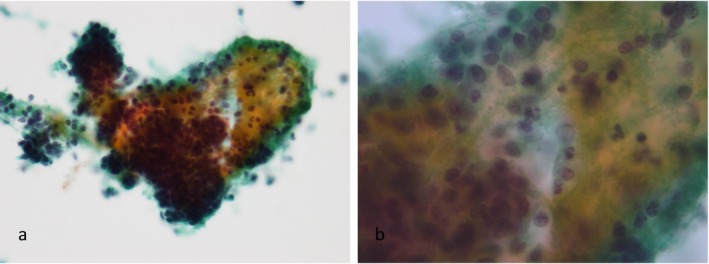
Endometrial glandular and stromal breakdown (EGBD): Fragmented epithelial cell clusters were detected. There were many condensed endometrial stromal cells accompanied by epithelial cell clusters that exhibited cellular changes (a, b). Light green bodies, which were considered to represent platelet‐induced thrombosis, were sometimes present. (a) ×200 magnification. (b) ×400 magnification.

**FIGURE 5:**
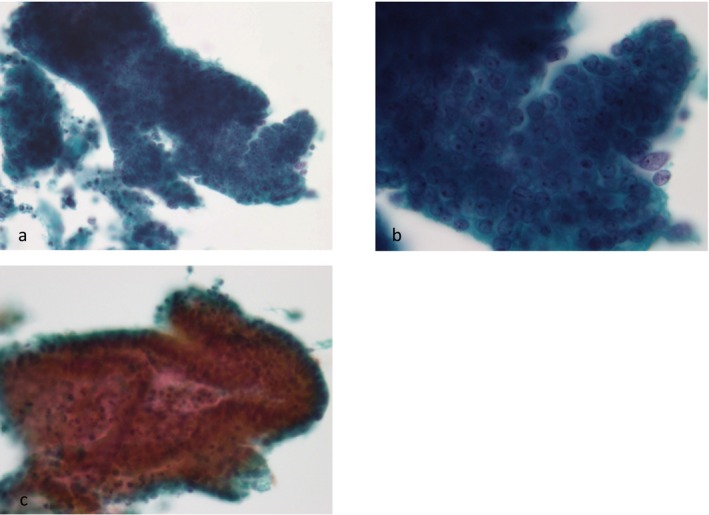
Endometrioid adenocarcinoma: The cell clusters in this condition exhibited more than five cell layers. Round and oval nuclei with nucleoli were detected, which are important for the differential diagnosis of this condition from EGBD (a, b, c). (a) ×200 magnification. (b) ×400 magnification. (c) ×200 magnification.

A summary of the diagnostic criteria in the Yokohama System is as follows: Traditional criteria, such as nuclear atypia, necrotic background, loss of cellular polarity and abnormal cell clusters, are considered significant findings. When cell clusters in a sample exhibit normal tubular or sheet patterns accompanied by stromal cells, a diagnosis of proliferative, secretory or atrophic endometrium is made, depending on the characteristics of the cells (Figures 1, 2, 3). However, when more than five atypical cell clusters showing dilated or branched patterns are observed, endometrial hyperplasia is diagnosed. If cellular atypia is also present, atypical endometrial hyperplasia or malignancy is suspected. The presence of papillotubular cellular clusters is strongly suggestive of malignancy. When tubular or sheet‐like patterns are absent, cell clusters with over 50 cells, nuclear overlap and nuclear morphology are critical diagnostic factors (Figures 4 and 5) [6, 12, 13, 14, 15, 16, 17, 18, 19, 20, 21].

## 3 Results

### 3.1 Number of Cytology Samples Analysed

All 112 ThinPrep‐based endometrial cytology samples re‐evaluated in this study were judged by the examiners to be of appropriate quality. As expected, the specimens were standardised, uniformly smeared, and of high quality, with no issues in microscopic observation.

### 3.2 Cytologic Interpretation With Histological Diagnosis

The eight cytologic examiners independently produced a total of 728 valid cytology reports following the standardised criteria of the Yokohama System. In these reports, results of ATEC‐AE (atypical endometrial cells, cannot exclude EAH or malignancies), atypical endometrial hyperplasia and malignancies were categorised as positive, while negative for malignancy, ATEC‐US (atypical endometrial cells, of undetermined significance) and proliferative endometrium without atypia were categorised as negative. The final pathology or clinical course confirmed 20 positive cases, which included:

- Endometrial carcinoma G1: 10 cases
- Endometrial carcinoma G2: 1 case
- Endometrial carcinoma G3: 1 case
- Serous carcinoma: 5 cases
- Clear cell carcinoma: 1 case
- Carcinosarcoma: 1 case
- Squamous cell carcinoma (invasion from cervix): 1 case

The remaining 92 cases were categorised as negative for malignancy. When the cytology results matched the final pathology and/or clinical outcomes, they were considered true positives or true negatives. When they did not match, they were classified as false positives or false negatives. The sensitivity, specificity, positive predictive value, negative predictive value and their respective 95% confidence intervals are presented in Tables 1 and 2.

**TABLE 1:** Final assessment of cytology results based on pathology findings.

Cytology result evaluation	Number of cases
True positive (TP)	124
False positive (FP)	46
True negative (TN)	550
False negative (FN)	8
Total	728

**TABLE 2:** Sensitivity, specificity, positive predictive value and negative predictive value of ThinPrep endometrial cytology with 95% confidence intervals.

	%	95% confidence interval
Sensitivity	93.90%	89.8%–98.0%
Specificity	92.20%	90.1%–94.3%
Positive predictive value	72.90%	66.2%–79.6%
Negative predictive value	98.60%	97.6%–99.6%

## 4 Discussion

Endometrial cytology using ThinPrep is expected to be valuable for early detection of endometrial cancer, providing a less invasive and simpler sampling method compared with endometrial biopsy. A recent breakthrough in specimen preparation techniques for ThinPrep‐based endometrial cytology has dramatically improved specimen quality.

As demonstrated in this study, the new preparation method using ThinPrep produced stable, high‐quality specimens, which contributed to improved diagnostic accuracy. ThinPrep‐based endometrial cytology prepared with this novel method is suitable for both clinical and research applications.

Comparing the results of this study to other studies on LBC methods, such as the prospective study using SurePath by Hirai et al. (Tables 3 and 4) [3], the sensitivity for detecting positive cases in this study (98.60%) was similar to that of SurePath and suction endometrial biopsy, with no significant differences. The specificity, however, was slightly lower at 92.20%, compared with SurePath and biopsy results. The reasons for this lower specificity, whether due to sample quality or evaluator inexperience, remain unclear.

**TABLE 3:** Sensitivity, specificity, positive predictive value and negative predictive value of SurePath endometrial cytology with 95% confidence intervals: cited from [3].

	%	95% confidence interval
Sensitivity	92.20%	85.7%–96.4%
Specificity	98.50%	97.5%–99.2%
Positive predictive value	87.60%	80.4%–92.9%
Negative predictive value	99.10%	98.3%–99.6%

**TABLE 4:** Sensitivity, specificity, positive predictive value and negative predictive value of suction biopsy pathology results with 95% confidence intervals: cited from [3].

	%	95% confidence interval
Sensitivity	85.20%	77.7%–91.0%
Specificity	98.90%	98.0%–99.5%
Positive predictive value	91.20%	84.5%–95.7%
Negative predictive value	98.10%	97.0%–98.8%

The positive predictive value (72.90%) was also lower compared with SurePath and biopsy results, possibly due to the same factors affecting specificity. Nevertheless, the negative predictive value (98.60%) remained high, indicating that this method effectively ruled out false negatives.

Although some of the diagnostic accuracy metrics of this study were slightly lower than those of studies using SurePath by experienced cytologic examiners, ThinPrep‐based endometrial cytology still showed sufficient accuracy for clinical application [22]. Furthermore, even examiners with little experience using ThinPrep were able to achieve relatively high diagnostic accuracy, suggesting that this method can be easily introduced globally. Its widespread adoption may contribute significantly to the early detection of endometrial cancer in clinical practice.

## 5 Conclusion

The novel preparation method using ThinPrep successfully provided high‐quality, standardised specimens. Furthermore, employing the Yokohama System enabled high accuracy in detecting endometrial malignancies, even for examiners with minimal experience with this cytology technique. This suggests that ThinPrep‐based endometrial cytology can be globally adopted with ease, potentially contributing significantly to the early detection of endometrial cancer.

## Author Contributions

Ms. Rie Ikemoto designed the study, collected references, analysed data and wrote the manuscript. Dr. Yasuo Hirai conceptualised, designed, supervised the study and revised the manuscript. All authors read and approved the final manuscript.

## Ethics Statement

This study was approved by the Research Ethics Committee of Yame public general hospital, Fukuoka, Japan.

## Conflicts of Interest

The authors declare no conflicts of interest.

## Data Availability

The datasets obtained and/or analysed in the current study are available from the corresponding author upon reasonable request.
